# Tβ4-exosome-loaded hemostatic and antibacterial hydrogel to improve vascular regeneration and modulate macrophage polarization for diabetic wound treatment

**DOI:** 10.1016/j.mtbio.2025.101585

**Published:** 2025-02-18

**Authors:** Hua Yu, Bin Wang, Zihao Li, Kaibo Liu, Wanying Chen, Songyun Zhao, Yu Zhou, Gaoyi Wang, Yaqin Zhou, Yanming Chen, Housheng Chen, Yunning Lai, Quan Wang, Jingping Wang, Binting Ni, Dupiao Zhang, Chuanmeng Pan, Yucang He, Liqun Li

**Affiliations:** a Department of Plastic Surgery, The First Affiliated Hospital of Wenzhou Medical University, Wenzhou, China; b Zhejiang Key Laboratory of Intelligent Cancer Biomarker Discovery and Translation, First Affiliated Hospital of Wenzhou Medical University, Wenzhou, China; c National Key Clinical Specialty (Wound Healing), The First Affiliated Hospital of Wenzhou Medical University, Wenzhou, China; d Department of Wound Healing, The First Affiliated Hospital of Wenzhou Medical University, Wenzhou, China

**Keywords:** Thymosin β4, Adipose-derived stem cells, Exosomes, Wound healing, Macrophage polarization, Angiogenesis

## Abstract

Diabetic wounds often exhibit delayed healing due to compromised vascular function and intensified inflammation. In this study, we overexpressed Thymosin β4 (Tβ4) in Adipose-Derived Stem Cells (ADSCs) to produce Exosomes (Exos) rich in Tβ4. We then utilized a dual photopolymerizable hydrogel composed of Hyaluronic Acid Methacryloyl (HAMA) and Poly-L-lysine Methacryloyl (PLMA) for the sustained release of Tβ4-Exos on diabetic wounds. The results showed that Tβ4-Exos could stimulate angiogenesis and collagen synthesis, and mitigate inflammation in diabetic wounds by promoting the polarization of M1-type macrophages and inhibiting that of M2-type macrophages. Furthermore, Tβ4-Exos was found to activate the PI3K/AKT/mTOR/HIF-1a signaling pathway, thereby enhancing vascular proliferation. In summary, the sustained release of Tβ4-Exos in HAMA-PLMA (HP) hydrogel and the management of inflammation through the upregulation of the HIF-1a pathway and modulation of macrophage polarization in vascular proliferation significantly accelerated the healing process of diabetic wounds.

## Graphical abstract

Scheme 1 **Schematic illustration of the synthesis method and therapeutic mechanism of Tβ4-Exos and HAMA-PLMA hydrogel.** (A) Application of Tβ4-Exos and HAMA-PLMA hydrogel on diabetic mice after synthesis; (B) HAMA-PLMA hydrogel loaded with Tβ4-Exos modulates macrophage M1 polarization towards M2, promotes angiogenesis, and increases collagen deposition; (C) Tβ4-Exos regulate HUVEC through the PI3K/AKT/mTOR/HIF-1a pathway. ADSCs: Adipose-Derived Stem Cells; Tβ4: Thymosinβ4; Exos: Exosomes; HAMA: Hyaluronic Acid Methacryloyl; PLMA: Poly-L-lysine Methacryloyl; HUVEC: Human Umbilical Vein Endothelial Cells. This figure was drawn by Figdraw.

**Figure:**
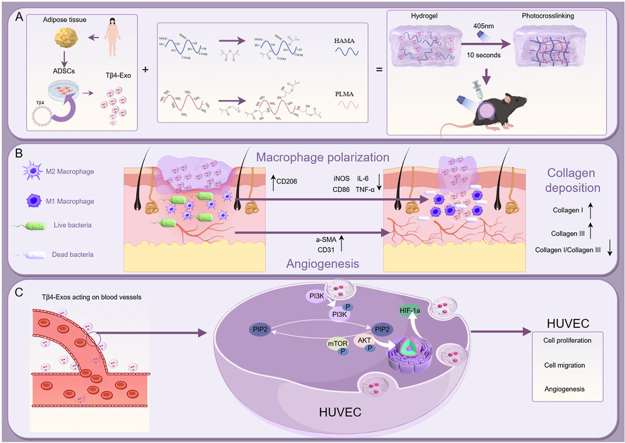

## 1 Introduction

Diabetic wound healing is a complex and significant health issue, affecting a substantial proportion of the global diabetic population. Annually, approximately 18.6 million diabetic patients worldwide develop foot ulcers, representing about 34 % of the total patient population. Notably, 20 % of these patients with diabetic foot ulcers undergo lower limb amputation, a procedure that is closely linked to increased mortality rates [1]. The delayed healing of diabetic skin ulcers is attributed to multiple factors, including impaired blood supply and exacerbated inflammation [2]. Additionally, a hallmark of diabetes is the exacerbation of microvascular damage due to chronic hyperglycemia [3,4]. Therefore, strategies to enhance angiogenesis and reduce inflammation are potential therapeutic approaches for diabetic wound healing.

Human Adipose-Derived Stem Cells (ADSCs) have been shown to promote wound healing [5], and the Exosomes (Exos) they secrete also facilitate this process [6]. ADSC-derived Exos offers several advantages over the stem cells themselves, such as reduced immunogenicity, increased safety, ease of storage and transportation, and the potential to act as molecular drug delivery vehicles [7]. Exos are known to promote wound healing through mechanisms including angiogenesis promotion and reduction of inflammatory responses [8]. In this study, to augment the beneficial effects of ADSC-Exos on wound healing, we employed lentiviral transduction technology to introduce the Tβ4 gene into ADSCs, resulting in Exos with high Tβ4 expression. Thymosin β4 (Tβ4), a 43-amino acid polypeptide from the thymopentin family, has been implicated in promoting angiogenesis and reducing inflammation in various studies: following myocardial cell injury, Tβ4, under the transcriptional regulation of ZEB2, increases its secretion, which promotes angiogenesis and mitigates cardiac damage [9]; similarly, Tβ4 can ameliorate the exacerbated inflammation resulting from LPS-induced inhibition of mitochondrial autophagy and passive activation of inflammasomes [10]; Tβ4 also regulates the activity of multipotent stem cells, alleviates cellular senescence, and participates in vascular construction in diabetic model mice [11]. During the process of wound healing, angiogenesis and reduction of inflammation are two important pathological and physiological processes, and studies have confirmed that Tβ4 promotes the healing of diabetic wounds [12]. Tβ4, as a peptide drug, has a short half-life, insufficient stability, and potential immunogenicity. To avoid the limitations of direct Tβ4 application, in this study, we utilized the characteristics of Exos, such as low immunogenicity, low toxicity, and the ability to be taken up by cells through various pathways including endocytosis and membrane fusion, to overexpress Tβ4 in Exos, thereby enhancing its therapeutic effects during the wound healing process.

Applying an antimicrobial hydrogel to the wound surface not only maintains wound moisture and cleanliness but also reduces the risk of infection. We developed a dual photopolymerizable hydrogel composed of Hyaluronic Acid Methacryloyl (HAMA) and Poly-L-lysine Methacryloyl (PLMA). This antimicrobial hydrogel is an optimal choice for wound dressings and, more importantly, serves as a carrier for Tβ4-Exos, prolonging their action in the wound and enhancing their functional effects. Hyaluronic acid, a key extracellular matrix component, is known for its high water retention [13] and maintains a stable cross-linked structure network, providing a three-dimensional porous structure akin to natural ECM [14], thus offering a continuously moist and biocompatible microenvironment for the wound suitable for drug delivery. ε-Polylysine (ε-PL), a naturally cationic antimicrobial peptide [15], can adsorb electrostatically to microbial cell surfaces, disrupt cell wall integrity, and inhibit various yeasts, Gram-positive, and Gram-negative bacteria [16]. The combination of HAMA and PLMA to form a hydrogel is primarily based on the network structure formed through cross-linking between them, which can effectively integrate the advantageous properties of both. This combination not only enhances the mechanical strength of the material but also improves its stability and functionality, offering more possibilities for applications such as drug controlled-release systems. We modified hyaluronic acid with methacryloyl groups and ε-PL with methacryloyl double bonds, endowing the HAMA-PLMA (HP) hydrogel with dual photopolymerizable rapid curing capabilities, which are beneficial for wound dressing formation, adhesion, hemostasis, and antimicrobial properties upon application.

In this study, we isolated primary ADSCs from the discarded fat of liposuction patients and overexpressed Tβ4, yielding Exos with high Tβ4 expression. To enhance the duration and efficacy of Tβ4-Exos, we designed an HP hydrogel with slow-release capabilities for Exos, along with injectability, adhesion, hemostasis, and good biocompatibility through the methacryloyl modification of hyaluronic acid and ε-polylysine. Our experimental results, including mouse wound blood flow detection, macrophage polarization marker detection, and endothelial cell transcriptome sequencing analysis, demonstrated that the HP hydrogel loaded with Tβ4-Exos can reduce inflammatory responses by affecting macrophage polarization and increase angiogenesis through the PI3K/AKT/mTOR/HIF-1a pathway, ultimately accelerating the healing of diabetic wounds.

## 2 Materials and methods

### 2.1 Primary extraction of Adipose-Derived Stem Cells (ADSCs) and exosomes (Exos)

This project has been supervised and approved by the Clinical Research Ethics Committee of the First Affiliated Hospital of Wenzhou Medical University. The ethical review number is KY2024-R202. Primary Extraction of ADSCs: Discarded adipose tissue was obtained from liposuction patients at our institution and processed by centrifugation at 1200 rpm for 5 min. The middle layer of adipose tissue was selected, while the remainder was discarded. An equal volume of Collagenase Type I solution (Sigma, CatNo. 9001-12-1) was added and the mixture was incubated on a 37 °C shaker for 1 h. Subsequently, an equal volume of serum-containing medium was added to quench the enzymatic activity. The digested tissue was then passed through a 70-μm sieve to obtain a single-cell suspension. After centrifugation at 1000 rpm for 5 min, the supernatant was aspirated, and the cell pellet was resuspended in a culture medium. The cells were plated in culture dishes and incubated at 37 °C in a humidified atmosphere with 5 % CO2. Fresh medium (Oricell, CatNo. HUXMD-90012) was supplied every three days to support cell expansion and subculturing.

Exosome Extraction: ADSCs from passages 3 to 5 were cultured in an Exosome-depleted complete medium for a minimum of 48 h, after which the supernatant was collected. Exosomes were isolated using a density gradient ultracentrifugation method. The supernatant was subjected to sequential centrifugation at 300*g*, 2000g, and 10000g for 30 min each, with the precipitate being discarded after each step and the supernatant carried forward. The remaining supernatant was then centrifuged at 120000g for 70 min twice, with the supernatant being discarded and the precipitate retained after each spin. The resulting Exosome-enriched precipitate was resuspended in PBS, quantified using the BCA protein assay, and stored at −80 °C for further analysis.

### 2.2 Testing of ADSCs differentiation ability

All induction differentiation media were procured from Oricell, Guangzhou, China. For adipogenic differentiation, 1 mL/well of 0.1 % gelatin was applied to six-well plates to ensure even coverage, left at room temperature for 30 min, and then the gelatin was removed by aspiration. Once the cell seeding achieved 100 % confluence in the six-well plates, culture medium A (CatNo. HUXMD-90031) was introduced for a 3-day induction period. This was followed by a 1-day maintenance period with medium B (CatNo. HUXMD-90031), after which medium A was reapplied for further induction. This cycle of alternating induction and maintenance was continued with daily monitoring of cellular morphology until the emergence of adequately sized lipid droplets, at which juncture Oil Red O staining was conducted and microscopic photographs were captured. For osteogenic differentiation, the same gelatin-coating procedure as described above was employed. Upon reaching 70 % confluence in the six-well plates, the cells were exposed to osteogenic induction differentiation medium (CatNo. HUXMD-90021), with fresh medium being supplied every 3 days. Following 2–4 weeks of induction, based on cellular morphological changes and growth, the cells were stained with Alizarin Red to assess mineralization. Chondrogenic differentiation followed a protocol analogous to that of osteogenic induction, with the exception that the medium was replaced with a chondrogenic induction differentiation medium (CatNo. HUXMD-90041).

### 2.3 Cell culture and cytological experiments

Human Umbilical Vein Endothelial Cells (HUVECs) and macrophage RAW264.7 cell lines were procured from the Chinese Academy of Sciences cell bank. HUVECs were maintained in an Endothelial Cell Medium (Sciencell, CatNo: 1001), while RAW264.7 cells were cultured in a specialized medium (Procell, CatNo.CM-0190). Both cell lines were grown under standard conditions of 37 °C in a 5 % CO2 incubator. The culture medium was formulated with 5.5 mM glucose to represent the normal glucose (NG) group, whereas the high glucose (HG) group contained 30 mM glucose. To achieve overexpression of Thymosin β4 (Tβ4) in Adipose-Derived Stem Cells (ADSCs), we employed lentiviral-mediated Tβ4 gene overexpression technology. Specifically, the lentiviral packaging kit (Genechem, Shanghai, China) was utilized according to the manufacturer's protocol to co-transfect the DNA-Lipofectamine2000 complex along with the target gene lentiviral vector into 293T tool cells. The resultant viral supernatant, after filtration, was collected for infecting ADSCs, with transfection efficiency confirmed through western blot and q-PCR analyses. The Tβ4 overexpression plasmid was synthesized using the following primer sequences: the upstream primer 5′-ACCGGTCGCCACCATGTCT-3′ and the downstream primer 5′-GCTAGCCGATTCGCCAGCT-3'.

### 2.4 Construction and characterization of HAMA-PLMA (HP) hydrogel

(HAMA)Hyaluronic Acid Methacryloyl (Cat No:EFL-HAMA-150K) was synthesized by introducing methacryloyl groups onto the hyaluronic acid molecule chain, a process conducted by Engineering for Life in Jiangsu, China. Simultaneously, (PLMA) Poly-L-lysine Methacryloyl (Cat No:EFL-PLMA- 001) was produced by modifying poly-L-lysine with methacryloyl groups through glycidyl methacrylate, thereby introducing double bonds onto the ε-poly-L-lysine molecule. To prevent the uncured hydrogel from obstructing the needle and to enhance its antibacterial capabilities, solutions of HAMA and PLMA freeze-dried powders were prepared at concentrations of 5 % w/v and 4 % w/v, respectively, following the manufacturer's guidelines. These solutions were made in a 0.25 % w/v initiator solution of Lithium Phenyl(2,4,6-trimethylbenzoyl) phosphinate and stirred at ambient temperature for a duration of 1 h. The solutions were then centrifuged at 4000 rpm for 15 min to eliminate bubbles. For sterilization, the hydrogel solution was heated to 80 °C for 30 min, followed by rapid cooling in an ice-water bath for 5 min, with this cycle being repeated once. The HP hydrogel solution was found to cure and adhere to the surfaces of wet instruments or tissues, such as the heart, liver, spleen, lungs, and kidneys, under 405 nm UV light within 10 s. To assess the cytotoxicity of the HP hydrogel, HUVEC or NIH 3T3 cells were cultured in 48-well plates with varying doses (0, 2, 4, 6, 8, and 10 μl) of the hydrogel. The CCK8 assay was employed to evaluate the hydrogel's impact on cell proliferation.

### 2.5 Exos sustained release experiment

This project has been supervised and approved by the Institutional Animal Care and Use Committee of the First Affiliated Hospital of Wenzhou Medical University. The ethical review number is WYYY-IACUC-AEC-2025-021. We selected male C57BL/6 mice at 10 weeks of age and created circular, full-thickness skin defect wounds with a diameter of approximately 1 cm on the dorsal region of the mice. A mixture containing DiR dye-labeled Exosomes (Exos) and hydrogel was locally administered onto the wound surface. In vivo imaging was conducted using the IVIS Spectrum system (PerkinElmer) at 1, 24, 48, 72, and 96 h post-injection. The excitation and emission wavelengths specific to the DiR dye were optimized within the imaging system to capture and quantitatively analyze images at the predetermined time points. Additionally, a similar mixture of DiR dye-labeled Exos and hydrogel in a 96-well plate was imaged and quantitatively analyzed at 6-h intervals throughout 6–48 h.

### 2.6 Mouse wound healing experiment and blood flow monitoring experiment

In the week preceding wound induction, male C57BL/6 mice aged 10 weeks were randomly selected to undergo diabetes modeling. After a 10-h fast, streptozotocin (STZ, 50 mg/kg) was intraperitoneally administered to induce type I diabetes. This treatment was continued for five consecutive days, and successful modeling was confirmed by a fasting blood glucose level exceeding 16.7 mmol/L (Supplementary Fig. 1), after which continuous blood glucose monitoring was initiated. Mice were anesthetized using pentobarbital sodium (50 mg/kg), the dorsal hair was shaved, and the skin was disinfected with an iodine solution. A circular skin incision of approximately 1 cm in diameter was created on the dorsal region of the mice. Wound photographs were captured on days 0, 2, 6, 10, and 14, and the wound areas were treated accordingly; the areas were measured using Image J software and statistically analyzed. To assess blood flow in the wounds, on the same days, mice were positioned laterally on the stage of a laser Doppler blood flow device, with adjustments made to achieve the optimal imaging plane. Blood flow imaging and quantitative analysis were performed using moorLDI ReviewV61 to determine the average blood flow values in the wound area.

### 2.7 Tissue immunofluorescence

After perfusion of the mouse heart, the wound tissue collected on day 14 was fixed, paraffin-embedded, and sectioned for histological analysis. The paraffin-embedded sections were subjected to deparaffinization and hydration, followed by antigen retrieval. The primary antibody was applied and incubated at 4 °C for 12 h, which was succeeded by a 1-h incubation at room temperature. Subsequently, the secondary antibody was added and incubated for 1 h at room temperature. The sections were then mounted with a fluorescence quenching agent containing 4′,6-diamidino-2-phenylindole (DAPI) to counterstain the nuclei. Photographs of the sections were captured using an inverted fluorescence microscope, and Image J software was utilized for subsequent statistical analysis.

### 2.8 Macrophage polarization flow cytometry

When RAW 264.7 cells in a 12-well plate reached approximately 40 % confluence, all groups were cultured in a medium containing 30 mM glucose (high glucose, HG) and 100 ng/mL lipopolysaccharide (LPS). The control, Exos, and Tβ4-Exos groups were subsequently incubated under hypoxic conditions for over 48 h. Post cell digestion, the cells were resuspended in phosphate-buffered saline (PBS) supplemented with 10 % fetal bovine serum (FBS) and incubated for 30 min within a 1.5 mL centrifuge tube. Following a wash with PBS containing 2 % FBS and centrifugation at 2000 rpm for 5 min, the supernatant was aspirated. A mixture of 3.6 μl of F4/80 antibody and 3.6 μl of CD86 antibody in 600 μl of PBS with 2 % FBS was used for further incubation in the dark for 45 min. After another centrifugation at 2000 rpm for 5 min and removal of the supernatant, the cell pellets were resuspended in 200 μl of PBS containing 2 % FBS per tube for flow cytometric analysis using a Beckman Coulter CytoFLEX instrument.

### 2.9 5-Ethynyl-20-deoxyuridine (EdU) assay

The experimental procedure was carried out in strict accordance with the protocol provided by the BeyoClick™ EdU kit (Beyotime, Shanghai, China). Initially, when the cellular monolayer in a 12-well plate achieved 60 % confluence, the cells were treated with a 10 μM EdU solution and co-incubated. Subsequently, the cells were fixed with a 4 % solution of polyformaldehyde for 30 min to preserve their morphology. This was followed by permeabilization with 0.3 % Triton X-100 for 10 min to facilitate antibody penetration. The cells were then subjected to staining with the click reaction mixture for 30 min to detect EdU incorporation. After a final counterstaining with DAPI for 10 min to visualize the nuclei, fluorescent images of the cells were acquired using an inverted fluorescence microscope.

### 2.10 Cell scratch assay

Upon reaching confluence, cells were subjected to a scratch wound assay. A sterile ruler was employed to guide the creation of vertical lines in the well plate using a 200 μl yellow pipette tip, which intersected with the bottom markings at several points, serving as fixed reference points for monitoring wound closure. Photographs were captured at various time intervals to assess the rate of scratch healing, and Image J software was utilized for the subsequent statistical analysis of these observations.

### 2.11 Transwell assay

A suspension of 4 × 10^4^ cells per well in 100 μl of serum-free medium was introduced into the upper compartment of the transwell apparatus (24-well plate, 8 μm pore size), while 500 μl of medium supplemented with 20 % serum was placed in the lower compartment. Following a 24-h incubation period, cells that had not migrated were removed from the upper chamber. The remaining cells were fixed with 100 μl of a 4 % polyformaldehyde solution for 15 min, after which they were stained with a Crystal violet staining solution (Biosharp, Shanghai, China) for an additional 15 min. The wells were then rinsed three times with distilled water for 5 min each to remove excess stains. Subsequently, the cells that had traversed the membrane were examined and quantified under a light microscope.

### 2.12 Tube formation assay

The GelNest™ basement membrane matrix (NEST, Wuxi, China, CatNo: 211212) was retrieved from −20 °C storage and allowed to thaw at 4 °C for 12 h. Subsequently, 30 μl of Matrigel was carefully applied to each well of a 96-well plate, ensuring that the entire well bottom was uniformly covered and free of air bubbles. The matrix was then allowed to solidify at 37 °C for 30 min. Cells were seeded into the wells at a concentration of 3000 cells per well and incubated for 6 h to permit the formation of tube-like structures. These structures were subsequently observed and photographed using an inverted microscope. Finally, Image J software was employed for the analysis and statistical processing of the images obtained.

### 2.13 RNA-seq experiment and data analysis

Total RNA was extracted from HUVECs that had been treated with varying concentrations of Tβ4-Exos and Exos, utilizing Trizol reagent as per the manufacturer's protocol. Subsequently, transcriptome sequencing was conducted by BGI Genomics (Shenzhen, China) on the DNBSEQ platform, with the project identified by the number F24A040006240_HOMbtijR. BGI Genomics was also responsible for providing all subsequent analytical services related to the sequencing data.

### 2.14 Statistical analysis

Statistical analyses were conducted using GraphPad Prism version 7.0 software. For comparisons between the two groups, the Student's t-test was employed. When comparing more than two groups, a one-way ANOVA was utilized to assess statistical significance. Results were deemed statistically significant at the ∗P < 0.05 threshold.

### 2.15 Antibodies and primers

Antibodies and primers are shown in Supplementary Table 1.

## 3 Results

### 3.1 Characterization of ADSCs, Exos, and Tβ4-Exos

**Fig. 1:**
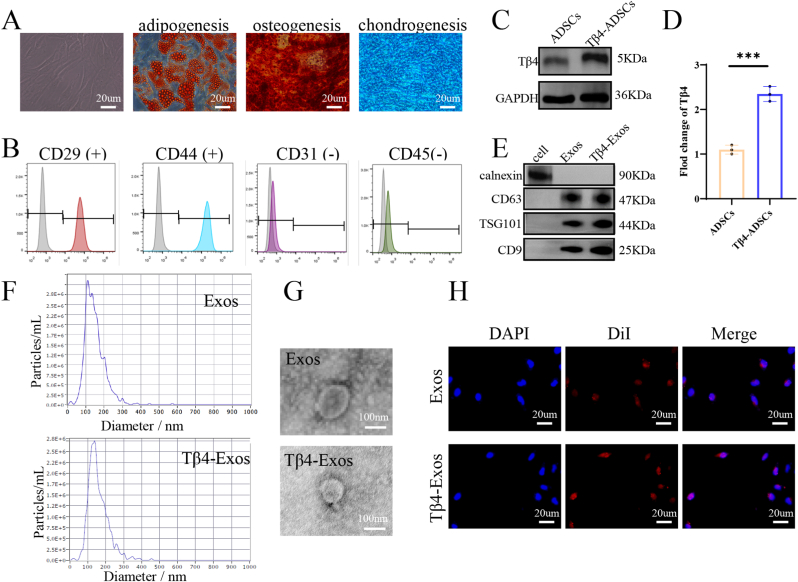
**Characterization of ADSCs, Exos, and Tβ4-Exos.**(A) Micrographs from an inverted phase contrast microscope depicted Adipose-Derived Stem Cells (ADSCs) exhibiting the characteristic spindle morphology and their capacity to differentiate into adipocytes, osteoblasts, and chondrocytes. (B) Flow cytometry confirmed the presence of positive surface markers CD29 and CD44, and the absence of negative surface markers CD31 and CD45 on ADSCs. (C, D) Western blot analysis was utilized to assess the expression levels of Thymosin β4 (Tβ4) in both ADSCs and Tβ4-transduced ADSCs. (E) Western blot analysis identified the exosomal markers CD63, TSG101, and CD9, and the absence of calnexin, confirming the exosomal nature of the samples. (F) Nanoparticle Tracking Analysis (NTA) was employed to quantify both Exosomes (Exos) and Tβ4-Exos. (G) Transmission Electron Microscopy (TEM) provided high-resolution images of Exos and Tβ4-Exos, revealing their morphological features. (H) Cellular fluorescence microscopy demonstrated the internalization of DiI-labeled exosomes (red) by HUVECs. "ADSCs" denote Adipose-Derived Stem Cells, and exosomes denote exosomes; P < 0.001 indicates statistical significance.

We successfully isolated Adipose-Derived Stem Cells (ADSCs) and conducted a series of characterization experiments. ADSCs displayed a characteristic spindle-shaped morphology under optical microscopy and exhibited the potential to differentiate into adipocytes, osteocytes, and chondrocytes, indicative of their stem cell properties (Fig. 1A). Flow cytometry analysis revealed that ADSCs expressed the surface markers CD29 and CD44, while lacking CD31 and CD45, aligning with the phenotypic profile of stem cells (Fig. 1B). Thymosin beta4 (Tβ4) was introduced into ADSCs through lentiviral transduction, and western blot analysis confirmed the efficiency of this transfection (Fig. 1C and D). Exosomes (Exos) were isolated from the supernatant of ADSCs by ultracentrifugation, and their identity was confirmed by the presence of positive markers (CD63, TSG101, CD9) and the absence of the negative marker calnexin, as detected by western blot, demonstrating that both Exos and Tβ4-Exos conformed to the expected exosomal characteristics (Fig. 1E). The size distribution of Exos and Tβ4-Exos, as determined by Nanoparticle Tracking Analysis (NTA), peaked at approximately 150 nm (Fig. 1F). Furthermore, both Exos and Tβ4-Exos presented the typical spherical cup-shaped morphology of membrane vesicles when observed under Transmission Electron Microscopy (TEM) (Fig. 1G). The cellular internalization of Exos was demonstrated by labeling them with DiI fluorescence dye, which showed their uptake by Human Umbilical Vein Endothelial Cells (HUVECs) (Fig. 1H). These characterization experiments confirmed that the overexpression of Tβ4 did not affect the morphology, appearance, or cellular uptake function of Exos.

### 3.2 Characterization and hemostatic performance of HAMA-PLMA (HP) hydrogel

**Fig. 2:**
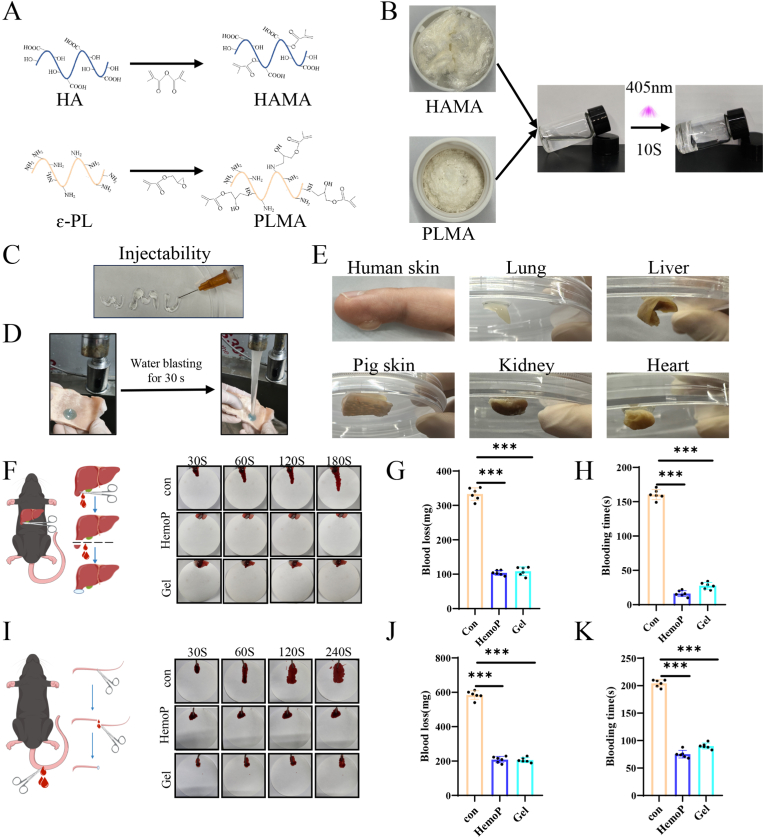
**Characterization and hemostatic performance of HAMA-PLMA (HP) hydrogel.** (A) Schematic representations of the molecular structures of Hyaluronic Acid Methacryloyl (HAMA) and Poly-L-lysine Methacryloyl (PLMA). (B) Schematic illustration depicting the formation process of the HAMA-PLMA (HP) hydrogel upon the mixing of HAMA and PLMA. (C) Assessment of the injectability of the HP hydrogel. (D) Evaluation of the HP hydrogel's resistance to water flow impact. (E) Examination of the HP hydrogel's adhesion properties. (F) Schematic diagrams and experimental setup for hemostasis models following mouse liver resection. (G, H) Quantitative analysis of blood loss and hemostasis time in the liver resection hemostasis model. (I) Schematic diagrams and experimental setup for hemostasis models following mouse tail amputation. (J, K) Quantitative analysis of blood loss and hemostasis time in the mouse tail amputation hemostasis model. "con" denotes the control group; "HemoP" denotes Hemostatic powder; P < 0.001 indicates highly significant differences.

We have developed a hydrogel designed for advanced wound management, endowed with a combination of beneficial attributes: hemostatic properties, injectability, strong adhesion, biocompatibility, and antimicrobial effects. Notably, this hydrogel is capable of encapsulating and gradually releasing Tβ4-Exos, thereby prolonging their therapeutic impact on wounds and accelerating the healing process. The hydrogel is synthesized through the cross-linking of two components: Hyaluronic Acid Methacryloyl (HAMA), which incorporates methacryloyl groups into hyaluronic acid, and Poly-L-lysine Methacryloyl (PLMA), obtained by the methacryloyl modification of poly-L-lysine (ε-Polylysine, ε-PL) to introduce double bonds (Fig. 2A). The HP dual-crosslinked hydrogel provides a rapid, 10-s photopolymerization process upon mixing (Fig. 2B). Nuclear magnetic resonance hydrogen spectrum (1H NMR) analysis showed that the appearance of proton peaks at chemical shifts of 6.18 ppm and 5.77 ppm in the PLMA spectrum (Supplementary Fig. 2A), and the appearance of proton peaks at chemical shifts of 6.11 ppm and 5.68 ppm in the HAMA spectrum (Supplementary Fig. 2B) indicated the successful grafting of the methacrylate groups in these two polymers. Scanning electron microscopy observations confirmed the formation of a uniform, porous network structure with pore sizes ranging from 30 to 160 μm inside the HAMA-PLMA hydrogel (Supplementary Fig. 3A), which provided an ideal site for loading Exos. Time-dependent rheological experiments showed that after UV irradiation, the gel underwent a transition from liquid to solid state, and its mechanical properties were significantly enhanced (Supplementary Fig. 3B). In the exosome release capacity test of HAMA-PLMA hydrogel, we found that 5 % HAMA +4 % PLMA had the best exosome release capacity (Supplementary Fig. 3C). Taking all these factors into account, we chose 5 % HAMA +4 % PLMA for all subsequent experiments. We used lap shear tests to verify the adhesion and strength of the HAMA-PLMA hydrogel, which showed good wet adhesion compared with the commercially available wound dressing Hydrosorb Gel (Supplementary Fig. 3D). HAMA-PLMA did not corrode or degrade in PBS solution for 2 weeks; however, it degraded by about 85 % in hyaluronidase solution naturally present in the body within 60 h (Supplementary Figs. 3E and F). To further evaluate the degradation performance of HAMA-PLMA, we subcutaneously injected 100uL of HAMA-PLMA into the back of mice. After sacrificing the mice on day 0 and day 7, the subcutaneous hydrogel was observed and photographed (Supplementary Fig. 3G). The results showed that the hydrogel seemed to have degraded mostly by day 7. This also explained that the release mechanism of Exos from the HAMA-PLMA hydrogel was the slow release of Exos through the continuous degradation of the hydrogel itself.

Further characterization of the hydrogel's physicochemical properties revealed its excellent injectability (Fig. 2C), its ability to adhere tenaciously to porcine skin even under a strong water jet for 30 s (Fig. 2D), and its high adhesion to wet surfaces of human skin, pig skin, and the moist surfaces of mouse heart, lung, kidney, and liver, without inducing allergic reactions or leaving any residue on the experimenter, a critical feature for biomedical applications (Fig. 2E). In both a mouse liver partial resection hemostasis model and a tail amputation hemostasis model, the HP hydrogel demonstrated hemostatic performance equivalent to that of a commercial medical hemostatic powder. In the liver partial resection model, the mean blood loss and hemostasis time for the hemostatic powder group and hydrogel group were 103.7 mg and 108.3 mg, and 16.2s and 27.5s, respectively, markedly lower than the control group's 333.1 mg and 160.3s (Fig. 2F–H). In the tail amputation model, the mean blood loss and hemostasis time for the hemostatic powder group and hydrogel group were 208.5 mg and 205.7 mg, and 75.3s and 90.2s, respectively, also significantly lower than the control group's 584.2 mg and 204.3s (Fig. 2I–K). These results underscore the HP hydrogel's robust hemostatic capabilities.

### 3.3 The HP hydrogel's excellent Exos sustained release capability

**Fig. 3:**
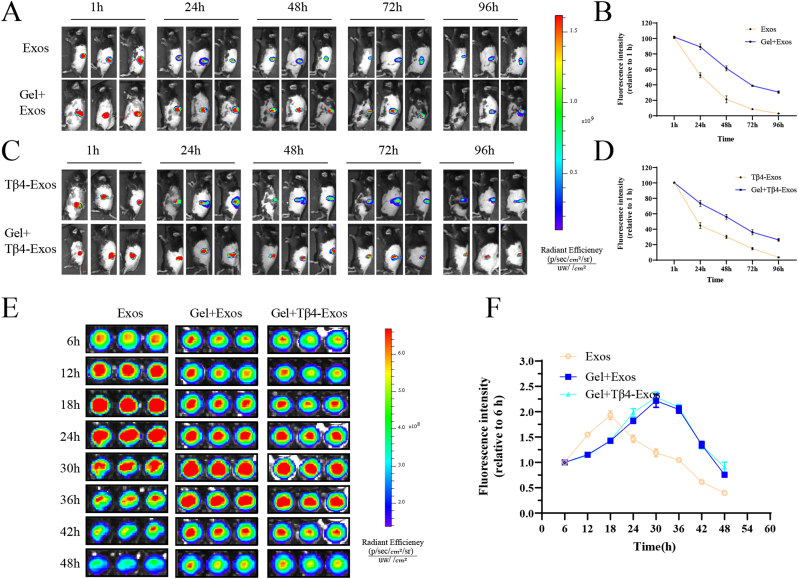
**The HP hydrogel demonstrated excellent exosome sustained-release capabilities.** In vivo, the HP hydrogel loaded with Exos was utilized for live imaging experiments, and the corresponding curves of exosome fluorescence intensity over time were recorded (A and B). Similarly, the HP hydrogel loaded with Tβ4-Exos was employed for live imaging studies, with the associated fluorescence intensity curves also documented (C and D). In 96-well plates, the In Vivo Imaging System (IVIS) was used to assess the release profiles of HP hydrogel-encapsulated Exos and Tβ4-Exos, with corresponding fluorescence intensity curves plotted over time (E and F). Statistical significance was determined at the P < 0.001 level.

To ascertain the hydrogel's efficacy in the sustained release of Exos, we performed in vivo optical imaging experiments using DiR dye-labeled Exos to track the release kinetics of Exos and Tβ4-Exos from the HP hydrogel. In these in vivo studies, the mean percentage of Exos remaining in the mouse wound after 48 h was 21.3 % for the Exos-only group and 61.3 % for the Gel + Exos group (Fig. 3A and B). Correspondingly, for the Tβ4-Exos group and the Gel + Tβ4-Exos group, the mean percentages of remaining Exos were 30.2 % and 62.7 %, respectively (Fig. 3C and D). These findings suggest that the incorporation of Tβ4 does not compromise the HP hydrogel's ability to sustain the release of Exos. Consistent with these in vivo observations, in vitro release experiments in a 96-well plate revealed that the fluorescence intensity peak for both the Gel + Exos group and the Gel + Tβ4-Exos group occurred at around 30 h, markedly later than the peak at approximately 18 h for the Exos-only group (Fig. 3E and F). In the in vitro experiments, the BCA assay of exosome-derived proteins demonstrated that both Exos and Tβ4-Exos were slowly released by HP hydrogel (Supplementary Fig. 4). Collectively, these results demonstrate that the HP hydrogel is effective in providing a sustained release of both Exos and Tβ4-Exos, as evidenced by both in vivo and in vitro experiments.

### 3.4 The biocompatibility of HP hydrogel

**Fig. 4:**
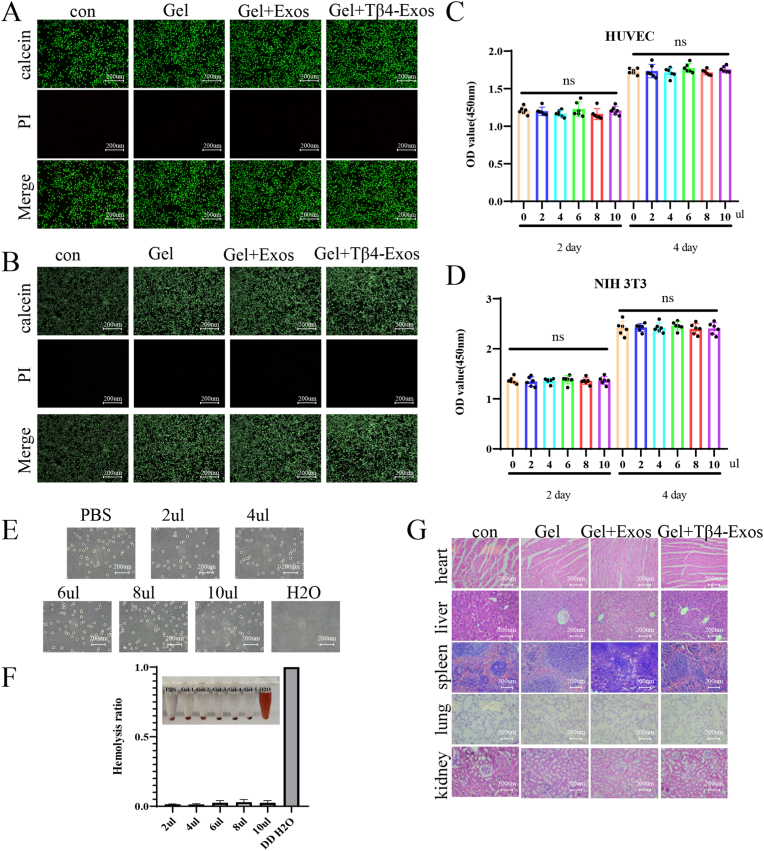
**Biocompatibility of HP hydrogel. Live-dead staining of Gel, Gel + Exos, Gel + Tβ4-Exos co-cultured with HUVEC cells.** (A) NIH 3T3 cells were utilized in the study. (B) CCK8 assays were conducted to evaluate the effects of hydrogel at varying concentrations when co-cultured with HUVEC cells (C) and NIH 3T3 cells (D). (E) Microscopic images and (F) corresponding hemolysis rates of hydrogel hemolysis experiments were captured at 400× magnification. (G) Pathological changes in the heart, liver, spleen, lungs, and kidneys of mice were assessed following the application of Gel, Gel + Exos, and Gel + Tβ4-Exos to wounds. "Con" denotes the control group.

In experiments utilizing live/dead cell staining, we evaluated the cytocompatibility of Gel + Exos, Gel + Tβ4-Exos, and their co-culture with HUVECs over 48 h, observing minimal red fluorescence indicative of dead cells (Fig. 4A). Comparable outcomes were noted in similar staining experiments with murine embryonic fibroblasts (NIH 3T3 cells) (Fig. 4B). We further assessed the impact of the HP hydrogel, added in increments from 2ul to 10ul in a 48-well plate, on HUVEC proliferation using the CCK8 assay. The findings indicated no significant alteration in cell proliferation rates in the presence of the HP hydrogel (Fig. 4C), and this was corroborated by results from NIH 3T3 cells (Fig. 4D). Collectively, these findings suggest that the HP hydrogel possesses excellent cytocompatibility. Additionally, in blood compatibility experiments with the HP hydrogel, microscopic observation revealed a plethora of intact biconcave disc-shaped red blood cells, with no signs of hemolysis (Fig. 4E). The hemolysis rate of the HP hydrogel across various concentrations remained below 5 % (Fig. 4F), which underscores the material's good blood compatibility. Simultaneously, no significant changes in hepatic and renal function parameters were observed in mice after administration across different groups (Supplementary Table 2). Furthermore, H&E staining of tissues from the Gel + Exos and Gel + Tβ4-Exos groups demonstrated no significant pathological alterations in the heart, liver, spleen, lungs, and kidneys, affirming the absence of substantial toxicity associated with the HP hydrogel (Fig. 4G).

### 3.5 Tβ4-Exos hydrogel accelerates wound healing in vivo

**Fig. 5:**
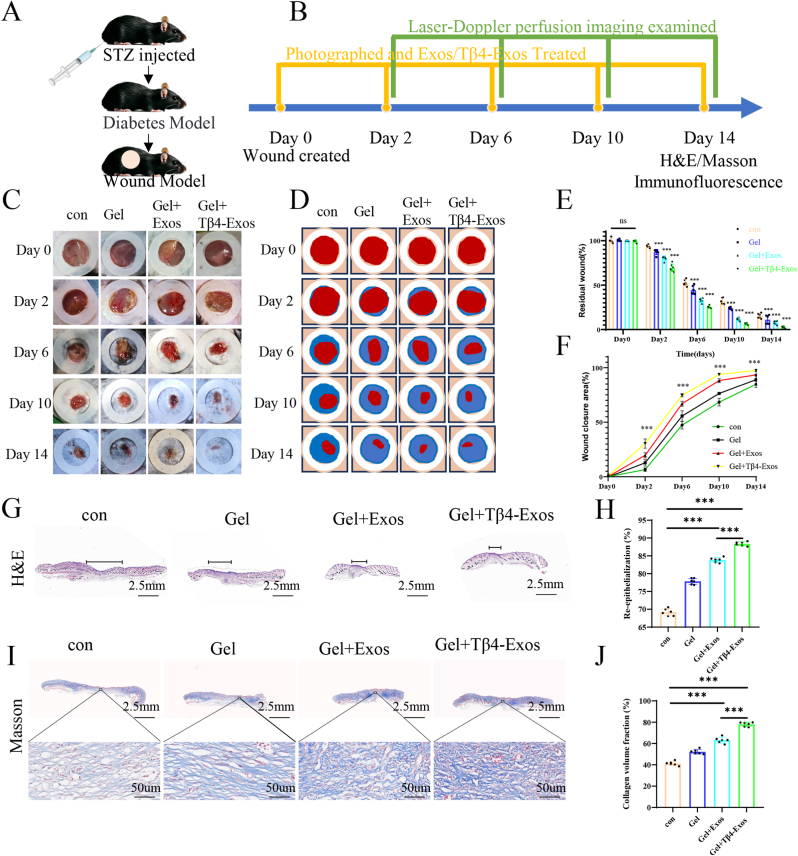
**Tβ4-Exos hydrogel accelerated wound healing in vivo.** (A, B) Schematic representations illustrate the modeling of diabetic wounds and the treatment schedule for the wounds. (C, D) Photographic documentation of the wound healing process in mice under various treatment regimens, alongside simulations depicting the area of wound closure. (E, F) Statistical evaluations of residual and healed wounds throughout the healing process in mice subjected to different treatments. (G, H) Histological images of H&E-stained wound samples on day 14, complemented by quantitative assessments of the re-epithelialization of the wound area. (I, J) Visual representations of Masson's trichrome-stained wound samples on day 14, along with quantitative analyses of the collagen content within the wound area. The term "con" refers to the control group; statistical significance is indicated at the P < 0.001 level.

To determine the efficacy of Tβ4-Exos hydrogel in accelerating the healing of diabetic wounds in vivo, we induced diabetes in C57 mice using Streptozotocin (STZ) and established a wound model on their dorsal regions. Various treatments were then administered from day 0 to day 14 (Fig. 5A and B). Macroscopic wound images revealed that wounds treated with Gel + Tβ4-Exos exhibited the most pronounced healing response compared to other treatment groups (Fig. 5C–F). On day 14, we collected wound tissue for H&E staining, which demonstrated that the Gel + Tβ4-Exos group had the shortest remaining wound length and the most effective healing (Fig. 5G and H). Additionally, Masson's trichrome staining was conducted on wounds treated with Gel + Tβ4-Exos, revealing thicker collagen fibers and a greater collagen area ratio (Fig. 5I and J).

### 3.6 Tβ4-Exos hydrogel promotes wound angiogenesis in vivo

**Fig. 6:**
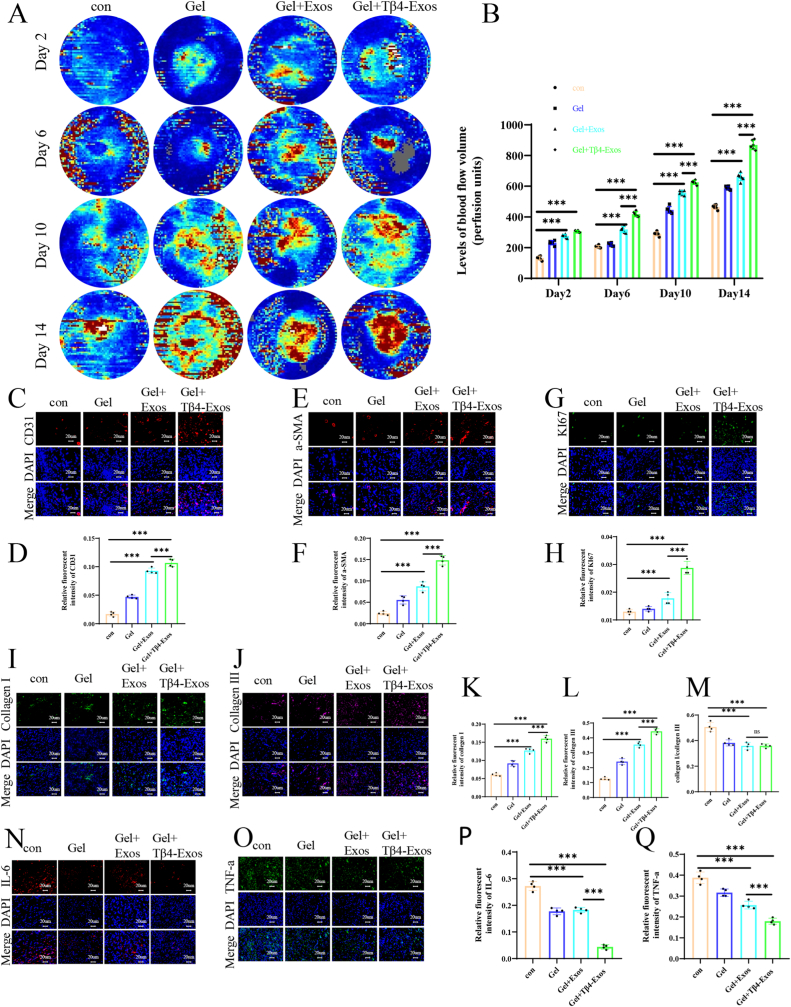
**Tβ4-Exos hydrogel promoted vascular generation, and collagen deposition, and reduced inflammation in wounds.** Laser Doppler imaging was employed to capture blood flow in wounds, with corresponding quantitative analysis provided (A and B). Tissue fluorescence staining was utilized to assess neovascularization, with images and quantitative analysis for the markers CD31 (C and D) and α-SMA (E and F). Additionally, the proliferation marker KI67 was evaluated through tissue fluorescence staining and quantitative analysis (G and H). The presence of Collagen I and Collagen III in the wound tissue was evaluated, with their distribution being elucidated through respective fluorescence staining for Collagen I (I and K) and Collagen III (I and K), and further supported by quantitative analysis. A quantitative analysis of the ratio of Collagen I to Collagen III in tissue fluorescence was also conducted (M). Inflammatory factors were assessed through tissue fluorescence staining and quantitative analysis for IL-6 (N and P) and TNF-α (O and Q). The control group is denoted by "con"; statistical significance is indicated at the P < 0.001 level.

We used a laser Doppler scanned images instrument to detect neovascularization and blood perfusion in the wounds, and the results showed that the average blood flow intensity in the wound area treated with Gel + Tβ4-Exos was the highest (Fig. 6A and B). Furthermore, in the immunofluorescence staining results of wound tissue at 14 days post-surgery, the Gel + Tβ4-Exos group had the highest expression of vascular neoformation markers CD31 (Fig. 6C, D) and α-SMA (Fig. 6E and F). Similarly, the proliferation marker KI67 in the Gel + Tβ4-Exos group was also the highest (Fig. 6G and H). These results showed that treatment with Gel + Tβ4-Exos enhanced angiogenesis in diabetic wounds, thereby promoting wound healing.

### 3.7 Tβ4-Exos hydrogel enhances collagen deposition in vivo

It is widely recognized that appropriate collagen deposition is crucial for the healing of diabetic wounds. We assessed the immunofluorescence staining of collagen I and collagen III in wound tissue at 14 days post-surgery and observed higher fluorescence intensity for both collagen I (Fig. 6I–K) and collagen III (Fig. 6J–L) in the Gel + Tβ4-Exos group. Furthermore, the ratio of collagen I to III was lower in the Gel + Tβ4-Exos group and Gel + Exos group compared to the control group (Fig. 6M), aligning with the principle that a higher proportion of collagen III is associated with scarless healing. The ratio of type I to type III collagen significantly influences scar formation [17]. Type I collagen primarily provides tissue strength and stability, while type III collagen plays a key role in regulating collagen fiber assembly and the flexibility of the extracellular matrix [18]. During wound healing, an increase in type III collagen can reduce the type I/type III collagen ratio, thereby decreasing scar formation. In various wound models, the content and distribution of type III collagen significantly affect the quality and speed of wound healing [19]. However, no significant difference was found between the Gel + Tβ4-Exos group and the Gel + Exos group, suggesting that Tβ4 may not contribute to scarless healing, whereas Exos may play a role in promoting this process. These findings indicate that the Tβ4-Exos hydrogel facilitated collagen deposition within the wound, contributing to scarless healing.

### 3.8 Tβ4-Exos hydrogel regulates macrophage polarization to reduce inflammation in wounds in vivo and in vitro

**Fig. 7:**
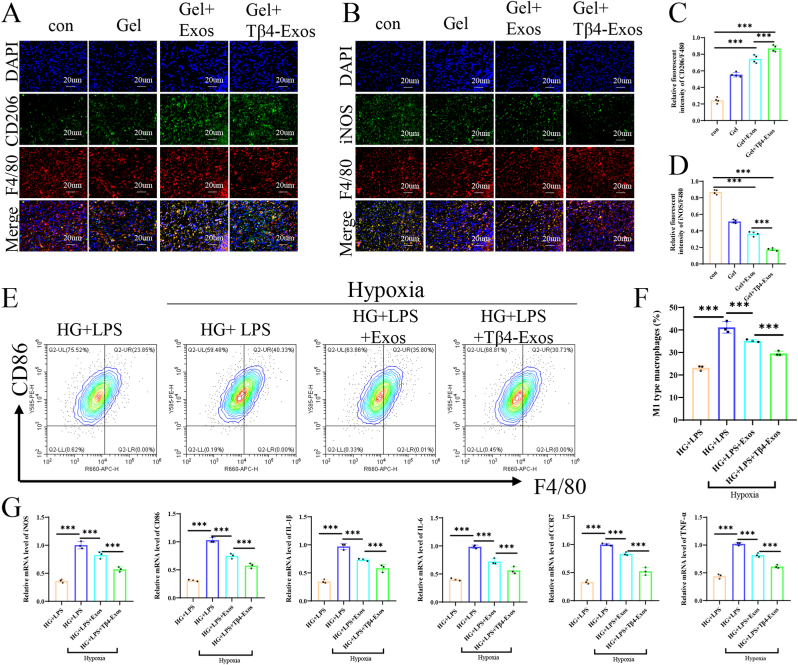
**The Tβ4-Exos-laden hydrogel modulated macrophage polarization, thereby attenuating inflammation in wound environments, as evidenced by both in vivo and in vitro studies.** (A, C) On day 14, dual immunofluorescence staining for F4/80 and CD206, indicative of M2 macrophage polarization, was performed, followed by quantification analysis. (B, D) Similarly, dual immunofluorescence staining for F4/80 and iNOS, markers of M1 macrophage polarization, was conducted on day 14, with subsequent quantification analysis. (E, F) Flow cytometry was utilized to detect the expression of CD86, an M1 macrophage marker. (G) Quantitative polymerase chain reaction (q-PCR) was employed to assess the expression of M1-associated markers, including iNOS, CD86, IL-1β, IL-6, CCR7, and TNF-α, following treatments across different groups. "HG" denotes high glucose conditions, while "con" signifies the control group; statistical significance is denoted at the P < 0.001 level.

Inflammation is a critical impediment to the wound healing process. We assessed the immunofluorescence staining of inflammatory markers, IL-6 and TNF-α, in wound tissue at 14 days post-surgery, finding higher fluorescence intensity in the Gel + Tβ4-Exos group for both IL-6 (Fig. 6N–P) and TNF-α (Fig. 6O–Q). These findings indicate that the Gel + Tβ4-Exos treatment effectively reduced the inflammatory response within the wounds. Further analysis of the wound tissue's immunofluorescence staining for pan-macrophage marker (F4/80), M2 marker (CD206), and M1 marker (iNOS) revealed that the CD206/F4/80 ratio was highest (Fig. 7A–C), and the iNOS/F4/80 ratio was lowest in the Gel + Tβ4-Exos group (Fig. 7B–D). This suggests that Gel + Tβ4-Exos promotes M2 polarization and inhibits M1 polarization of macrophages in vivo, thereby reducing inflammation and enhancing wound healing. In vitro, all RAW 264.7 cells were cultured in a medium containing 30 mM glucose (HG) and 100 ng/mL LPS to mimic the diabetic wound environment, and the control, Exos, and Tβ4-Exos groups were all subjected to hypoxic conditions to simulate the hypoxic microenvironment of diabetic wounds. Flow cytometry experiments detected the pan-macrophage marker (F4/80) and M1 marker (CD86), demonstrating that the proportion of M1 cells in the Tβ4-Exos group was significantly lower than in the control and Exos groups (Fig. 7E and F). Additionally, we measured the mRNA levels of M1-related markers (iNOS, CD86, IL-1β, IL-6, CCR7, TNF-α), and the results indicated that Tβ4-Exos treatment reduced the polarization of macrophages towards the M1 phenotype (Fig. 7G). Collectively, these results demonstrate that Tβ4-Exos modulates macrophage polarization by upregulating CD206 and downregulating iNOS, CD86, IL-6, TNF-α, and other inflammatory indicators, thereby reducing inflammation and promoting the healing of diabetic wounds.

### 3.9 Tβ4-Exos has been demonstrated to enhance cell proliferation, migration, and angiogenesis under high glucose conditions

**Fig. 8:**
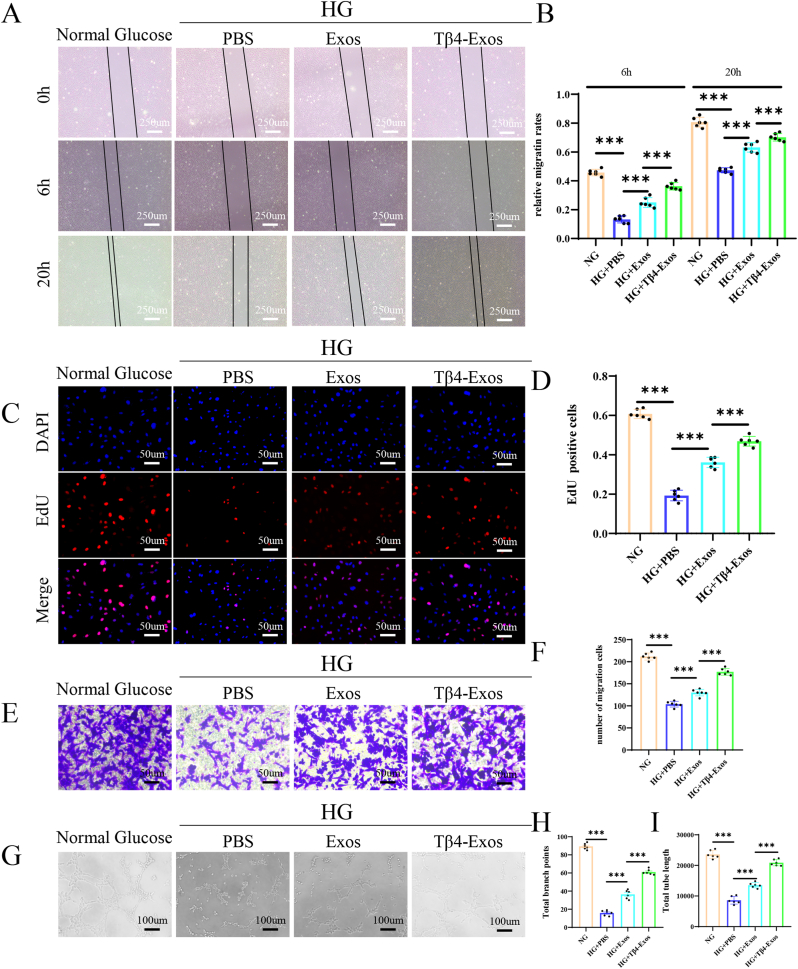
**Tβ4-Exos promoted cell proliferation, migration, and angiogenesis under high glucose conditions.** (A, B) Scratch assays were conducted to assess the migratory capabilities of different groups, complemented by quantification analysis. (C, D) EdU incorporation assays were utilized to evaluate cellular proliferation across various groups, with subsequent quantification analysis. (E, F) Transwell migration assays were performed to determine the migratory behavior of cells in different groups, followed by quantification analysis. (G, H, I) Tube formation assays were executed to assess angiogenic potential, with quantification analysis focusing on total branch points and total tube length for each group. "HG" indicates high glucose conditions; statistical significance is marked at the P < 0.001 level.

In vitro experiments substantiated the protective role of Tβ4-Exos on HUVECs subjected to high glucose stress, employing scratch assays, EdU assays, transwell migration assays, and tube formation assays. The scratch assay outcomes revealed that Tβ4-Exos significantly ameliorated high glucose-induced impairment (Fig. 8A and B). EdU assay findings indicated a marked increase in the proportion of EdU-positive cells under high glucose conditions with Tβ4-Exos treatment (Fig. 8C and D). Additionally, the transwell migration assay demonstrated a significant enhancement in cellular migration facilitated by Tβ4-Exos under high glucose conditions (Fig. 8E and F). The tube formation assay further disclosed that Tβ4-Exos augmented the number of branch points and total tube length under high glucose stimulation (Fig. 8G–I). Collectively, these results underscore the ability of Tβ4-Exos to stimulate cell proliferation, migration, and angiogenesis even in the presence of elevated glucose levels.

### 3.10 Tβ4-Exos promotes angiogenesis by activating the PI3K/AKT/mTOR/HIF-1a pathway

**Fig. 9:**
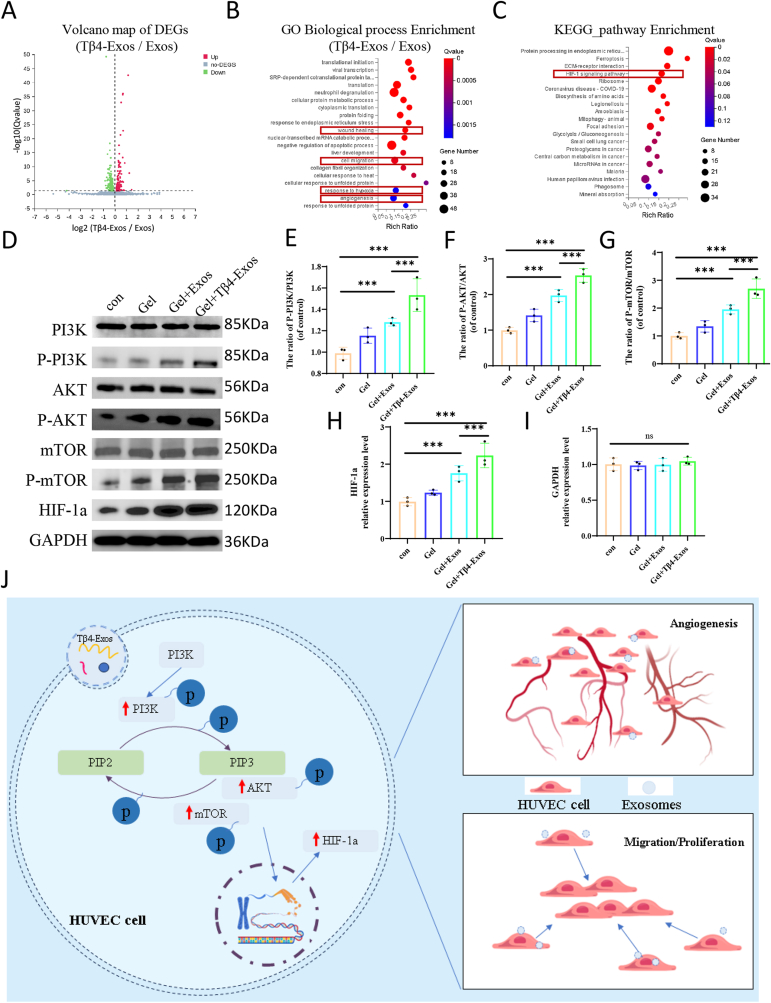
**Tβ4-Exos promoted the PI3K/AKT/mTOR/HIF-1a pathway leading to angiogenesis.** (A) Volcano plots were generated from transcriptome sequencing data to illustrate the differential expression of genes in the Tβ4-Exos group relative to the Exos group, highlighting both upregulated and downregulated genes. (B) A diagram depicting the results of Gene Ontology (GO) biological process enrichment analysis is presented. (C) A diagram of the Kyoto Encyclopedia of Genes and Genomes (KEGG) pathway enrichment analysis is shown. (D) Western blot analysis was performed to assess the protein expression levels of PI3K, phosphorylated PI3K (p-PI3K), AKT, phosphorylated AKT (p-AKT), mTOR, phosphorylated mTOR (p-mTOR), HIF-1α, and GAPDH. (E–I) Statistical summaries of the Western blot outcomes are provided. (J) A schematic representation illustrates how Tβ4-Exos activates the PI3K/AKT/mTOR/HIF-1α signaling pathway, which is crucial for endothelial cell proliferation, migration, and tube formation. "Con" denotes the control group; statistical significance is indicated at the P < 0.001 level.

Transcriptome sequencing analysis of HUVECs treated with Tβ4-Exos and Exos revealed that Tβ4-Exos induced the upregulation of 249 genes and the downregulation of 417 genes relative to Exos (Fig. 9A). These differentially expressed genes were significantly enriched in pathways associated with wound healing, cell migration, hypoxia response, and angiogenesis (Fig. 9B), aligning with our experimental observations (Fig. 8). Additionally, these genes showed a high level of enrichment in the HIF-1a signaling pathway (Fig. 9C). To elucidate the potential mechanism by which Tβ4-Exos enhances angiogenesis, we assessed the expression levels of PI3K/AKT/mTOR and its phosphorylated proteins, as well as HIF-1a in HUVECs under high glucose conditions using western blot analysis. The results indicated that Tβ4-Exos significantly elevated the levels of phosphorylated PI3K/AKT/mTOR proteins and increased HIF-1a expression (Fig. 9D–I). From these findings, we infer that Tβ4-Exos likely facilitates the migration, proliferation, and angiogenesis of diabetic wounds via the PI3K/AKT/mTOR/HIF-1a signaling pathway (Fig. 9J).

## 4 Discussion

The diabetic wound microenvironment, characterized by elevated glucose levels, is known to upregulate pro-inflammatory factors, exacerbate inflammation, and diminish angiogenesis, as referenced in the literature [20]. These sequential reactions impede the healing process of diabetic wounds. In this study, we successfully engineered ADSCs-Exos overexpressing Thymosin β4 (Tβ4) to facilitate the healing of diabetic wounds.

While stem cell transplantation offers the benefits of multilineage differentiation potential and certain immune-modulatory functions, it is not without significant challenges in clinical application, as documented [21]. These include the risk of immune rejection, technical hurdles, and the high costs associated with acquisition and preservation. An emerging body of research suggests that the therapeutic effects of stem cells within the human body are partially mediated through the paracrine release of Exos, rather than direct differentiation [22]. Exos possess anti-inflammatory properties and promote the polarization of M2 macrophages [23]. They offer higher safety in application and are more feasible in terms of production, storage, and transportation, rendering them more suitable for broad clinical use. Consequently, we selected ADSCs-Exos as the vector for Tβ4 overexpression.

With the advancement of biomaterials in tissue engineering and regenerative medicine, the continuous release and effective control of nanomaterials can intervene in diseases [24]. In the field of microenvironment-responsive nanomedicine, using biodegradable and biocompatible hyaluronic acid to encapsulate exosomes [25] is highly likely to continuously release exosomes for wound treatment as it degrades in the in vivo environment. However, the retention time of Exos within the body is not long, and they dissipate rapidly, requiring multiple administrations over a short period to maintain therapeutic effects [26]. Additionally, Exos have the drawback of low targeting efficiency. To overcome these challenges, it is crucial to develop hydrogel wound dressings that can provide sustained release of Exos, which can be directly applied to the surface of wounds to increase local concentration and duration of action, thereby enhancing the therapeutic efficacy of Exos. To address this issue, we developed a photo-curable, dual-crosslinked hydrogel composed of Hyaluronic Acid Methacryloyl (HAMA) and Poly-L-lysine Methacryloyl (PLMA). These hydrogels, with their pore size ideal for Exos loading, capitalize on the sustained release profile of Exos, enhancing both the potency and duration of Exos action. Moreover, leveraging the high water retention of hyaluronic acid and the natural antimicrobial properties of ε-poly-L-lysine (ε-PL), these hydrogels exhibit injectability, adhesion, robust hemostasis, antimicrobial activity, and good biocompatibility during the diabetic wound healing process. This material effectively mitigates various impediments to wound healing on multiple fronts.

This study also has certain limitations. For example, the challenges of exosome scaling include low separation and purification efficiency, difficulties in quality control and standardization, and limitations in cell culture and yield. The immune response that may be caused by lentivirus-transduced ADSCs is another limitation. Certain components on the surface of lentivirus vectors may be recognized by the immune system, thereby affecting transduction efficiency and ultimately impacting the stability of long-term gene expression. Therefore, in practical applications, we should optimize transduction conditions to reduce potential adverse effects on cells. The production cost of hydrogels, which is restricted by raw materials, production technology, quality control, and scaling up, also limits their large-scale production and wide application.

Thymosin β4 (Tβ4) is a highly conserved actin-binding peptide that plays a role in multiple biological processes, including the regulation of cytoskeletal dynamics [27], modulation of gene expression, and interaction with various signaling pathways to exert its pro-angiogenic and anti-inflammatory effects [28,29]. Under hypoxic conditions, Tβ4 facilitates the formation of new collateral vessels following myocardial infarction by modulating the miR-17-5p/PHD3/Hif-1α signaling pathway (PMC8964820). Additionally, synthetic dimeric Thymosin β4 (DTβ4) not only enhances the resistance of blood vessels to thrombosis but also stimulates the mobilization of CD93+/CD34+ cells, accelerating endothelial cell proliferation [30]. In this study, Tβ4-Exos increased the expression of vascular markers such as CD31 and α-SMA and further demonstrated the promotion of angiogenesis in mouse ultrasound Doppler blood flow detection experiments and in vivo HUVEC tube formation assays. Moreover, Tβ4, as an effective immune regulatory molecule, can guide the conversion of macrophages to the M2 phenotype and inhibit the fibrotic process, playing a positive role in bone repair [31]. This study demonstrated from various dimensions in vivo and in vitro that Tβ4-Exos promoted macrophage M2 polarization more than Exos and reduced M1 polarization.

Since the discovery of HIF in 1991, extensive research has yielded significant insights [[32], [33], [34]]. HIF-1 (hypoxia-inducible factor 1) is a crucial transcription factor for cellular adaptation to hypoxic conditions, enhancing our understanding of cellular responses to varying oxygen concentrations [35]. In promoting angiogenesis, the upregulation of PI3K/mTOR signaling can enhance HIF-α activity and induce the expression of angiogenic factors [36]. HIF-1α promotes angiogenesis by regulating the transcription of VEGF [37]. This study's transcriptome sequencing and western blot results confirmed that Tβ4-Exos activates the PI3K/AKT/mTOR/HIF-1a pathway, thereby promoting the migration, proliferation, and angiogenesis of diabetic wounds.

In summary, this study developed an injectable, hemostatic, antimicrobial, and long-acting HAMA-PLMA (HP) hydrogel for the sustained release of Tβ4-Exos. In a microenvironment mimicking diabetic wounds, the HP hydrogel loaded with Tβ4-Exos continuously exerted its effects on the wound surface, promoting the migration, proliferation, angiogenesis, collagen deposition, and vascular proliferation of diabetic wounds through the PI3K/AKT/mTOR/HIF-1a pathway, and modulating the M1-M2 transformation of macrophages to reduce inflammation, ultimately accelerating wound healing. This treatment model offers a promising strategy for the management of diabetic wounds.

## CRediT authorship contribution statement

**Hua Yu:** Writing – original draft, Visualization, Methodology, Investigation, Conceptualization. **Bin Wang:** Software, Methodology, Data curation. **Zihao Li:** Methodology, Investigation, Data curation. **Kaibo Liu:** Validation. **Wanying Chen:** Formal analysis. **Songyun Zhao:** Methodology. **Yu Zhou:** Software. **Gaoyi Wang:** Investigation. **Yaqin Zhou:** Data curation. **Yanming Chen:** Investigation. **Housheng Chen:** Methodology. **Yunning Lai:** Investigation. **Quan Wang:** Visualization. **Jingping Wang:** Visualization. **Binting Ni:** Visualization. **Dupiao Zhang:** Methodology. **Chuanmeng Pan:** Writing – review & editing, Validation, Supervision. **Yucang He:** Writing – review & editing, Validation, Supervision. **Liqun Li:** Writing – review & editing, Validation, Supervision, Resources, Project administration, Funding acquisition.

## Declaration of competing interest

The authors declare no conflicts of interest.

## Data Availability

Data will be made available on request.
