# Supplementary material for: Tβ4-exosome-loaded hemostatic and antibacterial hydrogel to improve vascular regeneration and modulate macrophage polarization for diabetic wound treatment
Source: Mater Today Bio. 2025 Feb 18;31:101585. doi: 10.1016/j.mtbio.2025.101585 (PMC11893380; doi:10.1016/j.mtbio.2025.101585)
Supplement: Multimedia component 1 [file mmc1.docx]

| Group | Non-diabetic control | Diabetic control | Gel | Gel+Exos | Gel+Lira-Exos |
| --- | --- | --- | --- | --- | --- |
| ALT（U/L） | 45.78±8.11 | 51.81±8.84 | 42.28±6.93 | 47.20±7.81 | 45.93±6.39 |
| AST（U/L） | 213.74±35.99 | 185.50±19.27 | 165.04±39.26 | 185.93±20.43 | 183.58±9.49 |
| BUN（mg/dL） | 19.30±0.66 | 22.05±2.13 | 20.01±1.45 | 17.44±.67 | 18.77±1.75 |
| Cr（umol/L） | 13.97±0.89 | 13.41±0.79 | 13.91±0.90 | 13.81±1.58 | 15.73±2.27 |

**
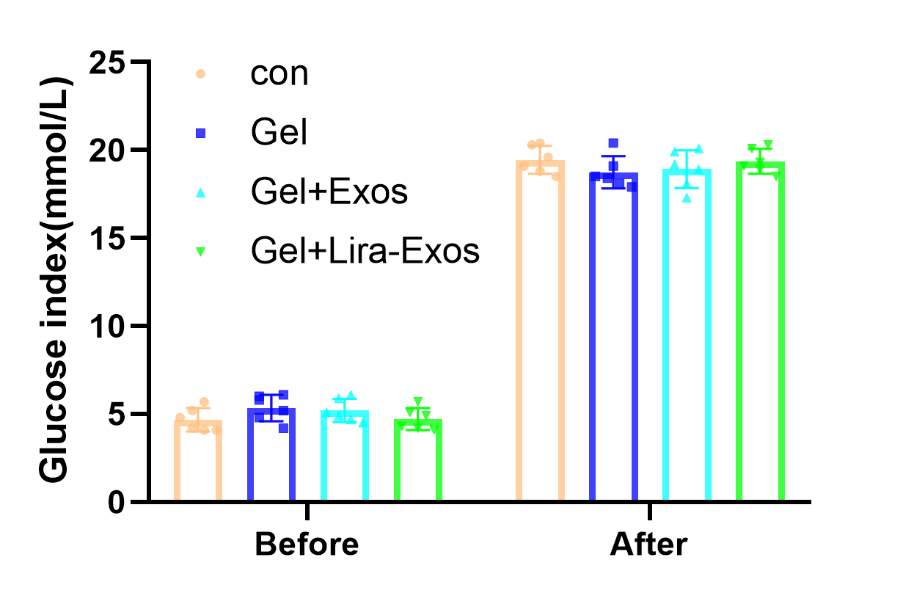
 Supplementary Figure 1.** Fasting blood glucose levels before and after diabetes modeling.

**Supplementary Table 2** Parameters of liver function and kidney function of mouse.

**
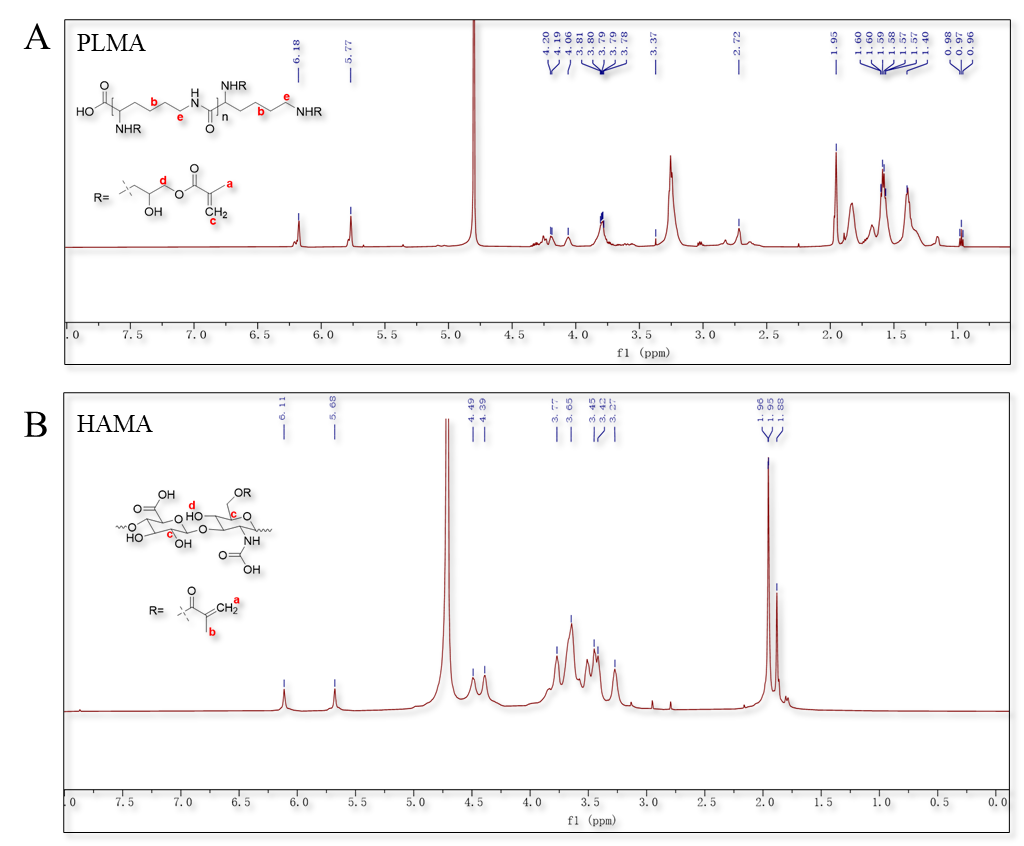
**

Supplementary Figure 2:The 1H NMR spectra of PLMA (A) and HAMA (B).

PLMA:1H NMR (600 MHz, D2O) δ 6.18, 5.77, 4.19, 4.06, 3.80, 3.37, 2.72, 1.95, 1.62, 1.55, 1.40, 0.97.

HAMA:1H NMR (600 MHz, D2O) δ 6.11, 5.68, 4.49, 4.39, 3.77, 3.65, 3.45, 3.42, 3.27, 1.98, 1.94, 1.88.

**
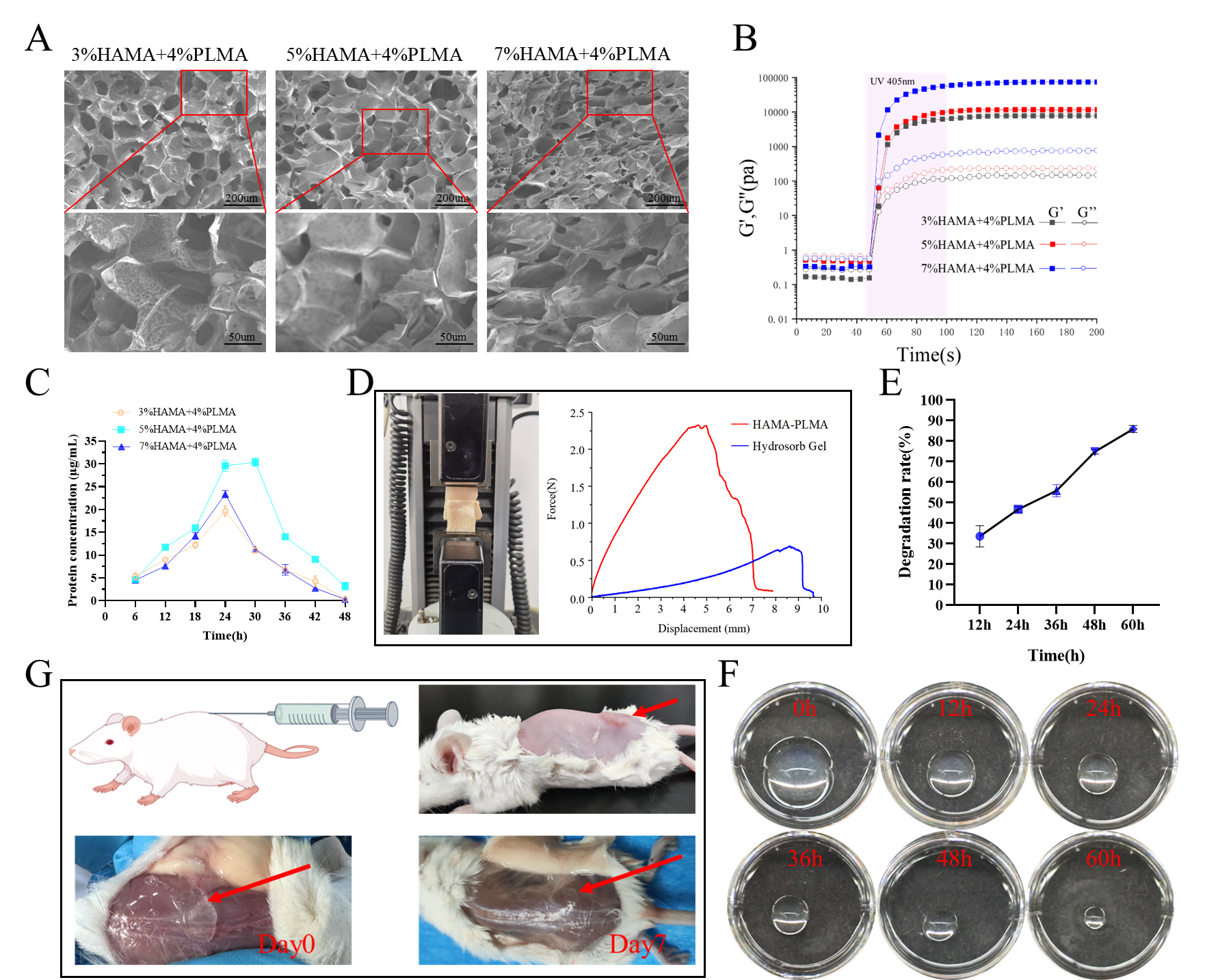
**

Supplementary Figure 3: Characterization data of HAMA-PLMA

(A) Scanning electron microscopy images of 3%HAMA+4%PLMA, 5%HAMA+4%PLMA, and 7%HAMA+4%PLMA; (B) Rheological property changes of 3%HAMA+4%PLMA, 5%HAMA+4%PLMA, and 7%HAMA+4%PLMA under UV irradiation conditions; (C) BCA assay of Exos-derived proteins from 1 ml of HAMA-PLMA mixed with 100ug/ml Exos soaked in 1 ml PBS at different time points; (D) Images of lap shear tests of HAMA-PLMA and Hydrosorb Gel (PAUL HARTMANN AG, Germany) adhered to pig skin; (E,F) In vitro degradation process and quantitative analysis of 100ul HAMA-PLMA in Hyaluronidase environment; (G) Schematic diagram of subcutaneous injection of HAMA-PLMA in vivo, and photos of HAMA-PLMA after subcutaneous injection on day 0 and day 7.

Supplementary Figure 4: BCA assay of Exos -derived proteins from 1 ml of HAMA-PLMA mixed with 100ug/ml Tβ4-Exos and Exos soaked in 1 ml PBS at different time points
